# A plausible role for actin gamma smooth muscle 2 (*ACTG2*) in small intestinal neuroendocrine tumorigenesis

**DOI:** 10.1186/s12902-016-0100-3

**Published:** 2016-04-23

**Authors:** Katarina Edfeldt, Per Hellman, Gunnar Westin, Peter Stalberg

**Affiliations:** Department of Surgical Sciences, Uppsala University, Uppsala University Hospital, Entrance 70, 1 tr, SE-75185 Uppsala, Sweden

**Keywords:** SI-NET, *ACTG2*, miR-145, Epigenetic regulation

## Abstract

**Background:**

Small intestinal neuroendocrine tumors (SI-NETs) originate from the enterochromaffin cells in the ileum and jejunum. The knowledge about genetic and epigenetic abnormalities is limited. Low mRNA expression levels of actin gamma smooth muscle 2 (*ACTG2*) have been demonstrated in metastases relative to primary SI-NETs. *ACTG2* and microRNA-145 (miR-145) are aberrantly expressed in other cancers and *ACTG2* can be induced by miR-145. The aim of this study was to investigate the role of *ACTG2* in small intestinal neuroendocrine tumorigenesis.

**Methods:**

Protein expression was analyzed in SI-NETs (*n* = 24) and in enterochromaffin cells by immunohistochemistry. The cell line CNDT2.5 was treated with the histone methyltransferase inhibitor 3-deazaneplanocin A (DZNep), the selective EZH2 inhibitor EPZ-6438, or 5-aza-2’-deoxycytidine, a DNA hypomethylating agent. Cells were transfected with *ACTG2* expression plasmid or miR-145. Western blotting analysis, quantitative RT-PCR, colony formation- and viability assays were performed. miR-145 expression levels were measured in tumors.

**Results:**

Eight primary tumors and two lymph node metastases displayed variable levels of positive staining. Fourteen SI-NETs and normal enterochromaffin cells stained negatively. Overexpression of *ACTG2* significantly inhibited CNDT2.5 cell growth. Treatment with DZNep or transfection with miR-145 induced *ACTG2* expression (>10-fold), but no effects were detected after treatment with EPZ-6438 or 5-aza-2’-deoxycytidine. DZNep also induced miR-145 expression. SI-NETs expressed relatively low levels of miR-145, with reduced expression in metastases compared to primary tumors.

**Conclusions:**

*ACTG2* is expressed in a fraction of SI-NETs, can inhibit cell growth in vitro, and is positively regulated by miR-145. Theoretical therapeutic strategies based on these results are discussed.

**Electronic supplementary material:**

The online version of this article (doi:10.1186/s12902-016-0100-3) contains supplementary material, which is available to authorized users.

## Background

Small intestinal neuroendocrine tumors (SI-NETs) are small, slow growing neoplasms that originate from the enterochromaffin cells in the ileum and jejunum. These rare tumors have an incidence about 1 case per 100 000. Metastases have often already occurred at time of diagnosis and the 5-year survival rate is around 65 %. Due to excess of tumor-secreted hormones; e.g. serotonin and tachykinins, patients can suffer from the carcinoid syndrome, causing cutaneous flushing, diarrhea, carcinoid heart disease and bronchoconstriction [1, 2]. The WHO classification from 2010 divides small intestinal neuroendocrine neoplasms in three grades; G1-NETs (Ki67 < 3 %), G2-NETs (Ki67 3–20 %) and NEC (neuroendocrine carinomas, Ki67 > 20 %) [3]. SI-NETs (G1 and G2) are most often resistant to chemotherapy and radiation, and medical treatment is limited. Symptom relief can be obtained by somatostatin-analogues and interferon treatment. There is a great need of new therapeutic options that could be beneficial to the patients.

The knowledge of common genetic or epigenetic abnormalities is limited in SI-NETs. Loss of chromosome 18 is most frequently seen, but no tumor-associated mutations have been found on chromosome 18 [4–6]. A putative role for *TCEB3C* (elongin A3), located at 18q21, as tumor suppressor gene in SI-NETs was recently suggested [7]. The mutation rate is overall low [8], and recently, exome- and genome sequencing found *CDKN1B* to be mutated in ~9 % of SI-NETs [9], implicating importance for this gene in tumorigenesis.

We have previously observed expression of actin gamma smooth muscle 2 (*ACTG2*) mRNA in a collection of primary SI-NETs, compared to undetectable expression levels in lymph node metastases [10]. Actin proteins are involved in multiple intracellular processes, including maintenance of the cytoskeleton and cell motility [11], and ACTG2 is normally found in enteric tissue. Aberrant expression has been described in several different cancer types and this can affect chemotherapy sensitivity [12–14]. Lower expression levels of *ACTG2* were detected in normal colon tissue compared to colon carcinoma [15]. High expression levels of *ACTG2* have been associated with improved disease-specific survival [16], and also with a more aggressive phenotype [17, 18]. Furthermore, microRNA-145 (miR-145) can positively regulate expression of *ACTG2* [19, 20], and overexpression of this microRNA inhibits cell proliferation, cell invasion, tumor growth and can induce apoptosis in other cancer cell types [19, 21].

The aim of this study was to investigate a possible role of *ACTG2* in small intestinal neuroendocrine tumorigenesis.

## Methods

### Tumor material and cell line

The patients included in the study (*n* = 28) were all diagnosed with SI-NET in the ileum and operated on at Uppsala University Hospital. This study was approved by the regional ethical review board in Uppsala (11-375/1.1.2011, Local ethical vetting board in Uppsala (Regionala etikprövningsnämnden i Uppsala)). Written informed consent for participation and publication of individual clinical details was obtained from all patients. All patients were above 18 years of age at time of inclusion. Fifteen tumors were classified as G1 NETs and 13 as G2 NETs. Patient characteristics are summarized in Additional file 1: Table S1. The tumors were snap frozen in liquid nitrogen and kept at −70 °C.

A SI-NET cell line, CNDT2.5, developed from a liver metastasis from a patient diagnosed with primary ileal SI-NET [22], was used in the experiments. These cells expressed neuroendocrine markers and somatostatin receptor 2 and responded to synthetic somatostatin analogue (octreotide) treatment [22, 23], although skepticism regarding the neuroendocrine authenticity of this cell line has also been raised [24]. The growth medium for CNDT2.5 was DMEM-F12 complemented with 10 % fetal bovine serum (Sigma Aldrich), 1 % vitamins, 1 % L-glutamine, 1 % sodium pyruvate, 1 % nonessential amino acids and 1 % PEST (penicillin-streptomycin), and the cells were cultured at 37 °C in 5 % CO_2_.

### Immunohistochemistry

Immunohistochemistry procedure is described in detail in previous research [25, 26]. Paraffin embedded tumor tissue (*n* = 24) sections (5 μm) were passed through descending alcohol concentrations and distilled water. Background staining was blocked with 3 % hydrogen peroxide and heated in citrate buffer. The tissues were treated with normal serum from goat (S-1000, Vector) and two different rabbit polyclonal anti-ACTG2 antibodies (NB100-91649 Novus Biologicals, diluted 1/200, and TA313418 Origene, diluted 1/80) and anti-chromogranin A antibodies (Ab-1, LK2H10, NeoMarkers, diluted 1/1000) were used and incubated. A biotinylated secondary antibody from goat anti-rabbit (BA-100 Vector, diluted 1/200) was added to the tissues and then treated with ABC complex. Visualization was done with DAB color reagent. Absence of primary antibody was used as a negative control. Consecutive sections from each tumor were incubated with anti-ACTG2 and anti-chromogranin A antibody. Also, consecutive sections of normal intestinal mucosa were treated with anti-ACTG2 (NB100-91649 Novus Biologicals, diluted 1/200) and anti-chromogranin A antibodies (Ab-1, LK2H10, NeoMarkers, diluted 1/1000).

### Immunofluorescence

Double immunofluorescence staining was done on sections of intestinal mucosa. Paraffin-embedded sections were deparaffinized, hydrated and subjected to pre-treatment (microwave heating for 10 min at 800 W, followed by 20 min at 450 W in citrate buffer, pH 6.0). The sections were blocked with normal goat serum (S-1000, Vector) for 30 min before incubation with primary antibody anti-chromogranin A (Ab-1, LK2H10, NeoMarkers, diluted 1/1000) for 90 min, followed by secondary antibody Alexa Fluor 488 goat anti-mouse for 30 min. Then, incubation with the next primary antibody anti-ACTG2 (NB100-91649 Novus Biologicals), for 90 min, was followed by the secondary antibody Alexa Fluor 594 goat anti-rabbit, for 30 min (Life Technologies). The sections were mounted with Vectashield with DAPI (Vector Laboratories Inc.) and evaluated under light microscope.

### Western blotting analysis

Proteins were extracted from tumors or CNDT2.5 cells using Cytobuster™ protein extraction reagent (Novagen) supplemented with Complete mini protease inhibitor cocktail tablets (Roche Diagnostics). Analysis of ACTG2 in tumor tissue was done using a primary antibody; anti-actin gamma2 (NB100-91649). Anti-Actin antibody (sc 1616, Santa Cruz) or coomassie blue was used as loading controls, and for verification of transfection results a mouse monoclonal anti-DDK antibody (TA50011, Origene) was used. After incubation with the appropriate secondary antibody, bands were visualized using the enhanced chemiluminescence system (GE Healthcare).

### Quantitative real-time RT-PCR

For extraction and purification of RNA, RNeasy Plus Mini Kit (Qiagen) was used according to manufacturer’s instructions, and for microRNA, miRNeasy Mini Kit (Qiagen) was used. Quantity was measured using NanoDrop. Reverse transcription of DNA-free RNA with random hexamer primers was performed using the “First strand cDNA Synthesis kit” according to manufacturer’s instructions (Fermentas) or MicroRNA RT kit (Life Technologies) using 10 ng RNA. Successful DNase I treatment of all RNA preparations was established by PCR analysis of the MYC promoter. qRT-PCR reactions were performed on the Step I qRT-PCR system (Applied Biosystems) using TaqMan Gene Expression Master Mix and assays for *ACTG2* (Hs00242273_m1), *GAPDH* (Hs02758991_g1), hsa-miR-145 (002278) and *RNU48* (001006) (Applied Biosystems). All samples were amplified in triplicates, and non-template controls were included. Each sample’s mean threshold value was corrected for the corresponding mean value for GAPDH mRNA or RNU48 miRNA, used as endogenous controls.

### Drug treatment

CNDT2.5 cells were seeded onto 6 well plates and treated with different concentrations of 5-aza-dC (5-aza-2’-deoxycytidine, Sigma Chemical Co., St. Louis, MO, USA, A3656) (0.025, 0.5, 1.0, 1.25, 1.5 μM) and DZNep (3-deazaneplanocin A, 2.5, 5.0, 10.0, 12.5, 15 μM) and cell viability was accessed using WST-1 (Roche Diagnostics GmbH). Not toxic concentrations were chosen; 1 μM for 5-aza-dC and 10 μM DZNep. Freshly prepared 5-aza-dC was used in the experiments. DZNep was kindly provided by Dr. Victor Marques [27].

2 × 10^5^ CNDT2.5 cells were seeded onto 6 well plates. After 24 h 10 μM DZNep or 1 μM 5-aza-dC was added in triplicates or 1, 2.5, or 5 μM EPZ-6438 (Selleckchem, Houston, TX, USA), a specific EZH2 inhibitor [28], was added to the wells and fresh medium and compounds were added every 24 h. The cells were harvested after 72 h, 96 h for EPZ-6438 treated cells, for RNA preparations. The DZNep treatment was repeated three times and 5-aza-dC and EPZ-6438 twice.

### miR-145 analysis

CNDT2.5 cells (1 × 10^5^) were distributed onto 6 well plates. After 24 h hsa-miR-145 or negative control miR (mirVana™miRNA mimics, Ambion) was transfected in triplicates using 20 mM miRNA and 8 μl INTERFERin siRNA transfection reagent (Polyplus Transfection). The cells were harvested and RNA prepared after 72 h. Transfections were repeated three times and successful transfection was determined by qRT-PCR using miR-145 assay. Apoptosis was measured in transfected cells using the Cell Death Detection ELISA kit (Roche Molecular Biochemicals), and as a positive control cells were incubated with 0.1 μg/ml Camptothecin (Sigma-Aldrich), a specific inhibitor of DNA topoisomerase I that induces apoptosis.

Frozen tumor sections from 24 tumors; 8 primary tumors, 9 lymph node metastases and 7 liver metastasis, were when needed macro-dissected to obtain at least 80 % tumor cells (in most cases more than 90 %) and RNA was extracted using TriZol reagent (Invitrogen), according to manufacturer’s instructions. cDNA synthesis followed by qRT-PCR was performed as described above.

### Proliferation and viability assays

A colony formation assay was performed and repeated three times; CNDT2.5 cells (1 × 10^5^) were seeded onto 6 well plates and transfected with 4 μg *ACTG2*-plasmid expression vector using 8 μl Lipofectamine 2000 reagent (Life Technologies) according to manufacturer’s instructions. The ACTG2 expression vector consisted of an expression-validated cDNA in pCMV6-Entry (TrueORF Gold, catalog no. RC203151. Origene Technologies, Inc., Rockville, MD, USA) and empty pcDNA3.1 was used as control. Six hours after transfection fresh medium was added complemented with 1 % PEST and 0.2 mg/ml Geneticin (G418, Sigma Aldrich). After 24 h 2000 cells were distributed onto 6 well plates and fresh medium with 0.2 mg/ml Geneticin was added every 72 h. After 8 days in selection the cells were fixed with 10 % acetic acid/10 % methanol, stained with 0.4 % crystal violet, and visible colonies were photographed and counted. Successful transfection was verified by western blotting after 24 h.

To analyze effect of *ACTG2* on viability, CNDT2.5 cells were transiently transfected and 1000 cells were seeded in a 96 well plate in triplicates. After 72 h cell viability was measured using the cell proliferation reagent WST-1 (Roche Diagnostics GmbH) according to manufacturer’s instructions.

### Statistical analysis

All data are presented as arithmetical mean ± standard deviation. Unpaired *t* test was used for statistical analysis and *p* < 0.05 was considered significant.

## Results

### ACTG2 protein is variably expressed in 42 % of analyzed SI-NETs

**Fig. 1:**
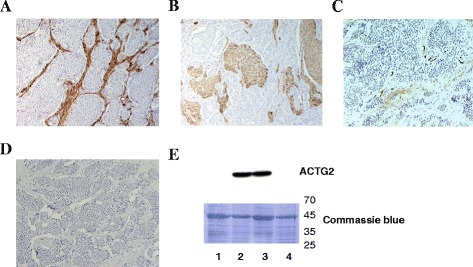
Analysis of ACTG2 protein expression in SI-NETs by immunohistochemistry (**a**-**d**) using ACTG2 antibody (NB100-91649 Novus Biologicals) and western blotting (**e**) using another ACTG2 antibody (TA313418 Origene). **a** Negatively stained tumor cells and strong stromal staining (20x). **b** Areas with positively stained tumor cells, and negative stromal staining (20x). **c** Weak staining in all tumor cells (20x). **d** No staining in absence of primary antibody (20x). **e** Western blotting analysis showing antibody specificity and correlation to immunohistochemistry analysis. One band only was visualized in two tumors (lanes 2 and 3) displaying strong stromal staining, and no band was detected in two tumors (lanes 1 and 4) with no staining in both tumor and stromal cells. Lane 1, lymph node metastasis; lanes 2–4, primary tumors. Coomassie blue staining was used as loading control, ladder in kDa

Protein expression was evaluated in 24 tumor sections from 17 patients; 16 primary tumors and 8 lymph node metastases (Additional file 1: Table S1). Fourteen tumors displayed no staining in the tumor cells (Fig. 1a) and six tumors were positive in small areas of the section (Fig. 1b). Furthermore, two tumors displayed larger areas of positive staining and two tumors were weakly positive in all of the tumor cells (Fig. 1c). No staining was detected in absence of primary antibody (Fig. 1d). In total, eight primary tumors and two lymph node metastases (10 out of 24; 42 %) displayed positive staining for ACTG2 in SI-NET cells (i.e. chromogranin-positive cells, data not shown). Connective tissue showed mostly positive staining in 19 tumors (Fig. 1a), four displayed mostly negative staining, and one tumor section lacked stromal tissue. A different anti-ACTG2 antibody was used and showed very similar results (data not shown). Western blotting analysis for ACTG2 revealed one band in the correct size range in two tumors with strong stromal staining and not in two tumors that showed negative immunohistochemical staining (Fig. 1e). No obvious relations of ACTG2 expression to clinical data were observed (not shown).

### ACTG2 protein is not detected in enterochromaffin cells of the normal small intestine

**Fig. 2:**
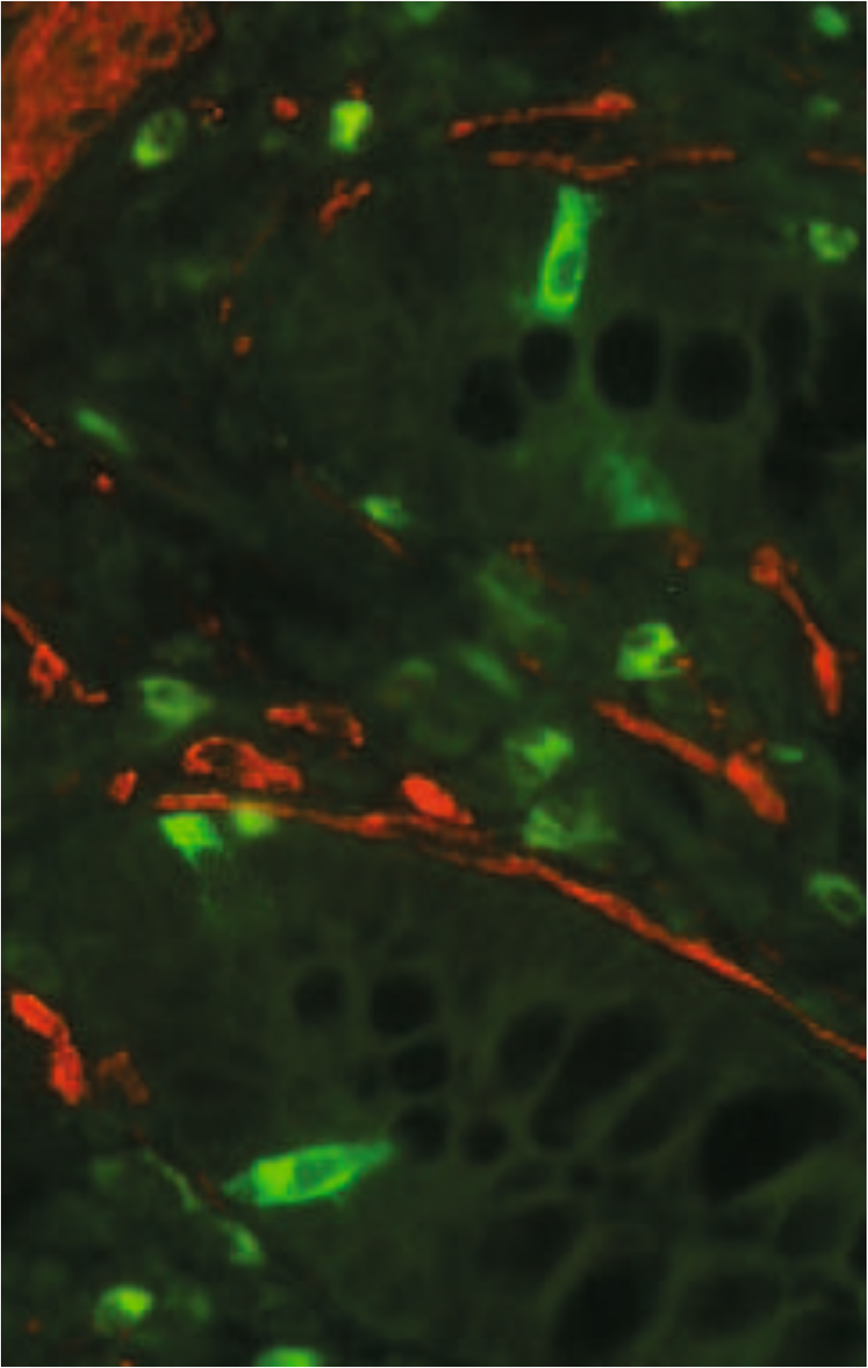
Double immunofluorescence staining of intestinal mucosa. Chromogranin A is visualized as green, showing positively stained enterochromaffin cells. ACTG2 is visualized as red and no staining is detected in chromogranin A positive cells (yellow)

In order to determine whether ACTG2 is expressed in chromogranin-positive cells of the normal intestinal mucosa (enterochromaffin cells), immunohistochemistry on consecutive tissue sections and also double immunofluorescence were performed. Thorough analysis did not reveal staining of both ACTG2 and chromogranin A in the same cell (Fig. 2). Since these enterochromaffin cells likely represent founder of SI-NET cells, our results suggest that ACTG2 expression can be induced by unknown mechanisms in a fraction of SI-NETs.

### *ACTG2* expression is induced by DZNep in vitro

**Fig. 3:**
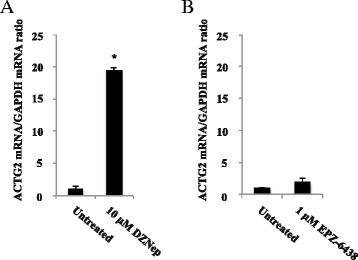
Effects on ACTG2 mRNA expression in CNDT2.5 cells after DZNep (3-deazaneplanocin A) and 1.0 μM EPZ-6438 treatment, **a** and **b** respectively

We next wondered whether *ACTG2* expression is controlled by epigenetic mechanisms and whether it could be induced by epigenetic drugs. Treatment of the human SI-NET cell line CNDT2.5, with the global histone methyltransferase inhibitor 3-deazaneplanocin A (DZNep) but not with the DNA hypomethylating agent 5-aza-2’-deoxycytidine, induced relative *ACTG2* mRNA expression approximately 20-fold (Fig. 3a; data not shown). However, this gene induction did not seem to involve the histone methyltransferase EZH2, which methylates histone 3 lysine 27 and is inhibited by DZNep, since treatment with the specific EZH2 inhibitor EPZ-6438 failed to induce *ACTG2* (Fig. 3b). Thus, *ACTG2* expression can be controlled directly or indirectly by mechanisms related to DZNep treatment, but other than EZH2 repression. It should be noted that positive controls for treatments with 5-aza-dC and EPZ-6438 were not included here.

### Involvement of miR-145

**Fig. 4:**
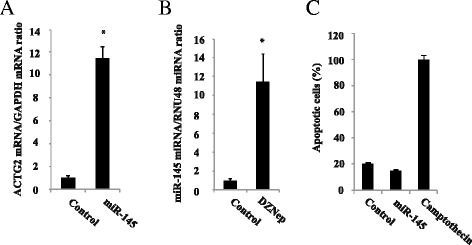
**a** Effects on ACTG2 mRNA expression in CNDT2.5 cells after miR-145 transfection. **b** Effects on miR-145 expression after DZNep treatment. **c** Quantitative determination of cytoplasmic histone-associated-DNA-fragments (mono- and oligonucleosomes) after miR-145 transfection. Camptothecin at 0.1 μg/ml was used as positive control

**Fig. 5:**
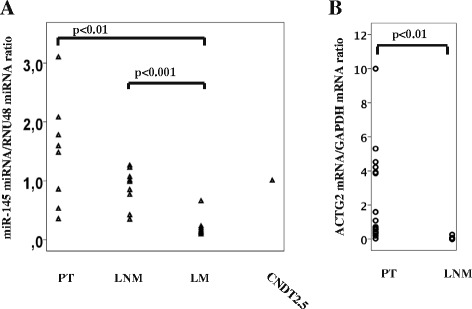
**a** miR-145 expression levels in 24 SI-NETs, and in CNDT2.5. A significant (*p* < 0.01) difference between primary tumors and liver metastases, and also between lymph node and liver metastasis (*p* < 0.001) was observed. A tendency towards decreased expression in lymph node metastases compared to primary tumors was detected (*p* = 0.09). **b** *ACTG2* mRNA expression levels in 18 PT and 16 LNM. A significant (*p* < 0.01) difference between primary tumors and lymph node metastases was observed. PT, primary tumor. LNM, lymph node metastasis. LM, liver metastasis

Expression of *ACTG2* is known to be positively regulated by miR-145 in other cell types, and this was also observed (~12-fold) in CNDT2.5 cells transiently transfected by miR-145 (Fig. 4a). The level of miR-145 was increased more than 1000-fold in transfected cells, as determined by quantitative RT-PCR (data not shown). Interestingly, the expression of miR-145 was induced by DZNep treatment (~11-fold) (Fig. 4b). miR-145 is known to induce apoptosis in other cell types, but this was not observed here (Fig. 4c). Relative miR-145 expression was then determined in 24 SI-NETs; with a mean threshold cycle (Ct)-value of 32.4 and somewhat higher expression in 5 primary tumors compared to metastases and CNDT2.5 cells. miR-145 was significantly less expressed in liver metastases compared to primary tumors (Fig. 5a). There was a tendency towards decreased expression in lymph node metastases compared to primary tumors (*p* = 0.09). This needs to be examined in a larger cohort, although in line with these results, previously published experiments have shown significantly reduced expression of *ACTG2* mRNA in lymph node metastases compared to primary tumors (Fig. 5b) [10].

### Growth inhibition by *ACTG2* in vitro

**Fig. 6:**
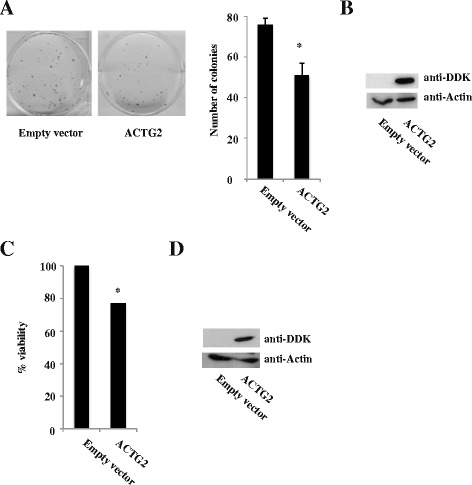
**a** Colony formation assay in CNDT2.5 cells stably transfected with a plasmid expressing ACTG2 or with empty expression vector. **b** Western blotting demonstrating successful transfection of the DDK epitope fused to ACTG2. **c** Viability assay using WST-1 after transient overexpression of ACTG2. **d** Western blotting demonstrating successful transfection of the DDK epitope fused to ACTG2

To investigate whether *ACTG2* could control SI-NET cell growth, a colony formation assay was performed on CNDT2.5 cells stably transfected with an *ACTG2* expression plasmid or empty vector. A significantly reduced ability to form colonies (by 32 %) compared to control cells was observed (Fig. 6a and b). This finding was supported by the reduced viability (Fig. 6c and d), supporting a growth inhibitory effect of *ACTG2* in vitro.

## Discussion

*ACTG2* is often aberrantly expressed in multiple cancers [15, 17, 18], and low levels have been associated with worse disease-specific survival [16]. Previously, low mRNA expression levels of *ACTG2* were demonstrated in metastases relatively to primary SI-NETs [10]. To investigate a possible role and function of *ACTG2* in SI-NET tumorigenesis this finding was first confirmed by immunohistochemistry, demonstrating absence of protein expression in the majority of investigated SI-NETs. Interestingly, eight primary tumors and two lymph node metastases displayed positive staining for ACTG2 in tumor cells, albeit at variable level and appearance. We could not detect ACTG2 expression in the enterochromaffin cells of the normal intestinal mucosa, suggesting that expression of *ACTG2* can be induced at some point during tumor progression representing a dedifferentiated phenotype, rather than being normally expressed in this cell type. Induction of *ACTG2* at some point during primary tumor growth may have beneficial effects as *ACTG2* showed growth inhibitory effects, at least in vitro. Expression of *ACTG2* was detected in stromal cells and whether ACTG2 can display growth effects here remains to be investigated.

This study demonstrated that expression of *ACTG2* can be induced by DZNep treatment or miR-145 transfection of the human SI-NET cell line CNDT2.5. Treatment with DZNep also induced expression of miR-145, indicating a possibility that induction of *ACTG2* by DZNep may be due to the effects on miR-145 expression. DZNep is a potential drug in cancer treatment [29]. DZNep can inhibit the histone methyltransferase EZH2, which is the catalytic subunit of polycomb repressive complex 2 and is responsible for methylation of lysine 27 on histone 3, a repressive mark [30]. A role of EZH2 was however excluded here since EPZ-6438, a newly developed specific drug inhibiting EZH2 enzymatic activity [28], was not able to induce *ACTG2* expression. MiR-145 is often deregulated in cancer cells [31, 32] and is known to induce *ACTG2* expression in breast cancer [19]. Here, it is demonstrated that this occurs also in SI-NET cells; overexpression of miR-145 increased expression of *ACTG2* in vitro*.* There was a decrease of miR-145 expression in metastasis compared to primary tumors, as observed for *ACTG2* [10]. Low levels of *ACTG2* are correlated to chemotherapy resistance [12, 14] and inducing this gene in SI-NETs would, not only have a growth inhibitory effect, but also potentially make the tumors more sensitive to treatment. SI-NETs are difficult to cure due to their resistance to chemotherapy and radiation, and new treatment strategies are warranted. MicroRNAs are involved in gene regulation and cancer development, and thus, have a potential role as therapeutic targets. miR-145 has been suggested to be a candidate for RNA medicine in colon tumors with a reduced expression [33]. miR-145 have multiple gene targets, and seems to be able to act as both a tumor suppressor and an oncogene depending on tumor type. Ruebel et al. [34] detected a difference in expression levels of miR-145 between primary SI-NETs and metastases, and here we confirmed a decrease in expression by tumor progression. These results suggest that miR-145 may be a tumor suppressor and may be important for the ability to metastasize. Inducing or introducing miR-145 may be a potential new therapeutic strategy in SI-NETs.

## Conclusions

Involvement of *ACTG2* in small intestinal neuroendocrine tumorigenensis has not been investigated previously. Here, we demonstrate that ACTG2 protein expression can be detected in a fraction of SI-NETs and absent in others, and that it is regulated by miR-145. Overexpression of *ACTG2* inhibited cell growth and reduced cell viability in vitro. Further investigation is needed to determine if introducing miR-145 in SI-NETs could have therapeutic advantages.

### Ethics and consent to participate statement

This study was approved by the regional ethical review board in Uppsala (11-375/1.1.2011, Local ethical vetting board in Uppsala (Regionala etikprövningsnämnden i Uppsala)). Written informed consent for participation and publication of individual clinical details was obtained from all patients.

### Consent to publish statements

Written informed consent for participation and publication of individual clinical details was obtained from all patients.

### Availability of data and materials statement

The data is presented in the main manuscript and in an Additional file 1: Table S1.

## Additional file

Additional file 1: Table S1. Patient characteristics, immunohistochemistry analysis of ACTG2 and miR-145 analysis in SI-NETs (XLSX 49 kb)
